# Development and evaluation of a digital simulation tool for teaching gynecological cytology: a pilot study with nursing students

**DOI:** 10.1186/s12909-026-09883-4

**Published:** 2026-07-09

**Authors:** Andressa Germano da Silva, André Luiz Brandão, Sandra Pereira Gama, Camilo Lellis-Santos, Andréa Cristina de Moraes Malinverni

**Affiliations:** 1 Department of Pathology, Universidade Federal de São Paulo, Pedro de Toledo Street, 781, 5th floor, São Paulo, SP CEP 04039-032 Brazil; 2 Center for Mathematics, Computation, and Cognition, Universidade Federal do ABC, Santo André, Brazil; 3 HUMAN Lab, INESC-ID and Instituto Superior Técnico, Universidade de Lisboa, Lisbon, Portugal; 4 Department of Biological Sciences, Universidade Federal de São Paulo, Diadema, Brazil

**Keywords:** Digital simulation, Nursing education, Gynecological cytology, Educational technology, Emotional engagement, Student perceptions

## Abstract

**Background:**

Training in gynecological cytology within nursing education presents challenges, including limited opportunities for practical experience and a lack of empirically evaluated educational tools. Digital simulation may offer a structured and safe approach to support learning, learner engagement, and practical preparation. This study aimed to develop and evaluate PapSim, a digital simulation tool for teaching gynecological cytology to nursing students.

**Methods:**

A pilot educational study was conducted with 40 second-year nursing students following a theoretical session on gynecological cytology. Participants engaged with the PapSim prototype in a supervised laboratory setting. Emotional engagement was assessed using a structured questionnaire based on the Pleasure Framework. Participants rated each emotional dimension on a six-point (0–5) ordinal response scale. Quantitative data were analyzed descriptively and through Spearman’s rank correlation analysis. Qualitative responses were examined using thematic content analysis. Results: Higher mean scores were observed in the categories of Simulation (4.63), Discovery (4.60), and Exploration (4.48), indicating strong engagement with immersive and task-oriented dimensions. Lower scores were reported in Danger (0.78) and Subversion (1.23), reflecting perceptions of safety and appropriateness. Significant positive correlations were identified between Discovery and Competition (*ρ* = 0.69, *p* < 0.001), as well as between sensory and empathic dimensions. Qualitative findings reinforced participants' perceptions of realism, cognitive stimulation, and spontaneous peer interaction during the simulation.

**Conclusions:**

The PapSim prototype was positively perceived and well accepted by nursing students, supporting its use as a complementary educational tool for teaching gynecological cytology. Further studies involving larger and more diverse samples are warranted to evaluate educational outcomes.

## Background

Cervical cancer remains a significant global public health challenge, particularly in low- and middle-income countries, where access to preventive measures is limited. According to the World Health Organization (World Health Organization, 2024), more than 600,000 new cases and over 340,000 deaths from cervical cancer occurred worldwide in 2020, with approximately 90% of these deaths occurring in low-resource settings [1]. Effective strategies such as HPV vaccination and cervical screening, including the Papanicolaou test, are critical to the early detection and prevention of cervical cancer and its precursor lesions.

Persistent infection with *Human Papillomavirus* (HPV), a sexually transmitted virus, is responsible for 99% of cervical cancer cases. Among the more than 200 genotypes of HPV identified, types 16 and 18 are considered high risk, accounting for approximately 70% of cervical cancer cases. On the other hand, low-risk types such as HPV 6 and 11, although they can cause benign lesions such as condylomata acuminata and laryngeal papillomas, have no oncogenic potential [2].

The integration of updated, technology-enhanced teaching resources in pathology education - aligned with national curricular frameworks for health education - is essential to ensure high-quality training for future health professionals. A lack of curricular standardization, coupled with limited practical training hours, can impair students’ preparedness to address the demands of healthcare systems [3]. In the field of gynecological cytology, the limited availability of empirically evaluated educational tools represents a critical gap; addressing this gap could enhance strategies for cervical cancer prevention and early diagnosis [4]. In this context, interactive educational tools such as games and simulators offer a promising approach by providing practical and engaging learning experiences that support more effective and context-based training in public health.

The use of digital educational simulators emerges as a promising approach to addressing training gaps in health education, particularly in gynecological cytology. These tools provide a practical, accessible, and immersive approach that enhances students’ understanding of key concepts related to the Papanicolaou test, HPV, and primary prevention strategies. In addition to supporting technical learning, interactive technologies help raise students’ critical awareness of reproductive health and highlight the role of nursing in promoting health equity [4].

This study aimed to develop and evaluate a functional prototype of a digital simulator designed to teach gynecological cytology to nursing students. The initiative seeks to offer an interactive educational tool that supports practical and autonomous learning, covering all stages of the process - from sample collection to cytological slide analysis. The goal is to provide an interactive educational resource that supports learning and familiarization with the stages of gynecological cytology, contributing to nursing education in reproductive health [5]. Additionally, because digital simulations often fail to address learners’ emotional responses, this study adopts the Pleasure Framework to explore learners’ emotional engagement and the acceptability of the tool. Understanding students’ affective reactions can help identify motivational, social, and perceptual factors that enhance the adoption and pedagogical impact of simulation-based education. This approach is particularly relevant in nursing education, where emotional and empathic dimensions play an important role in learning and student engagement.

## Methods

### Ethical approval

The study was approved by the institutional Research Ethics Committee in accordance with national ethical guidelines for research involving human participants (CAAE 75286923.0.0000.5505). All students who participated in the simulation signed the Informed Consent Form (ICF), ensuring voluntary participation in compliance with ethical standards.

### Setting

The activity involved second-year nursing students (Class 85) from UNIFESP. Of the 62 students enrolled in the course, 40 chose to voluntarily participate in the simulation experience after signing the ICF.

### Simulator setting and sampling

**Fig. 1:**
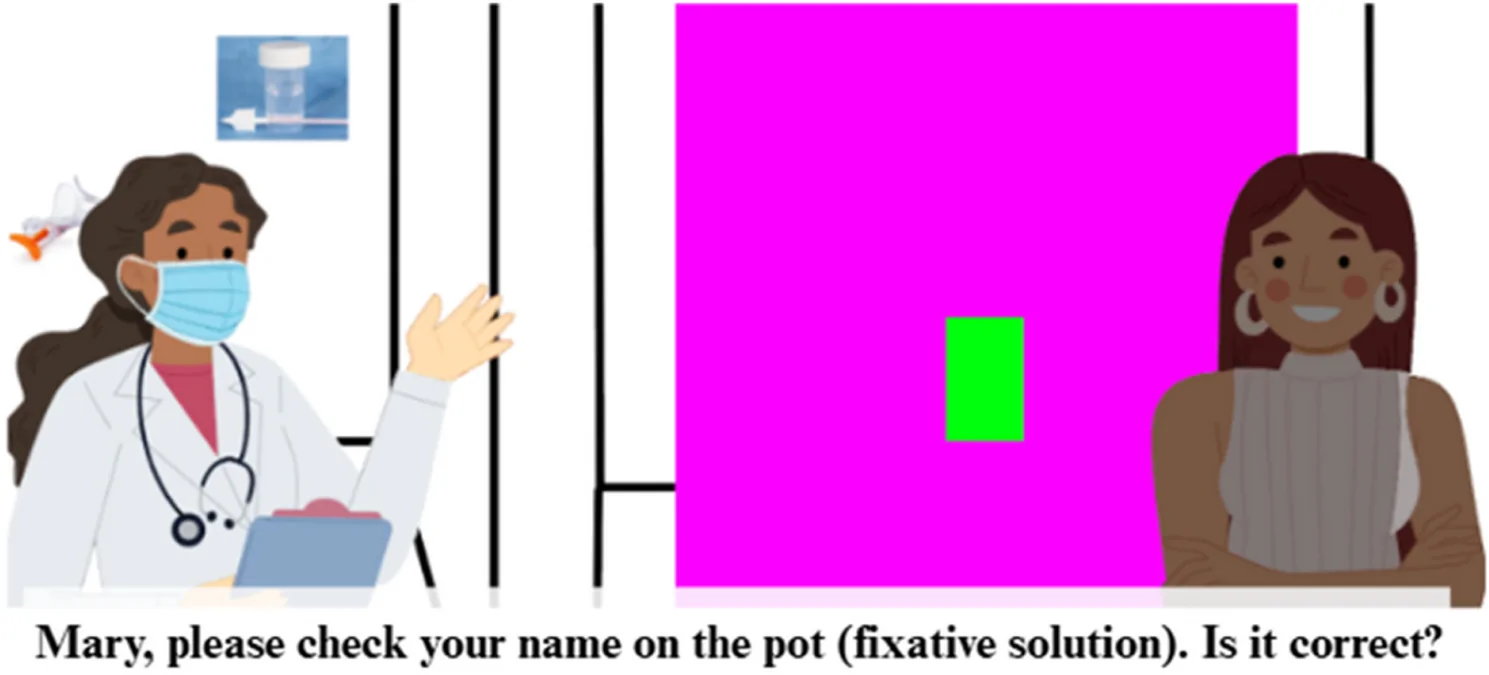
Scene 1 of the PapSim Simulator Prototype. Nurse Florence (Left) asks the patient Mary (Right) to check if her name is correctly labeled on the vial containing the fixative solution. This step precedes the cervical cytology collection procedure, ensuring safety and traceability. At this prototype stage, the simulator is available in Portuguese

The simulation was conducted in the university’s computer laboratory, which housed 27 computers. To organize access to the PapSim digital simulator, participants were randomly divided into two groups: one with 27 students and another with 13. This allowed full use of the laboratory infrastructure and minimized overcrowding. The PapSim simulator was developed using the free version of the Unity development platform, programmed in C#. Its design was preceded by the creation of a storyboard, which guided the structuring of the interactive scenes. The simulator is divided into two sequential scenes: the first takes place in a sample collection room, where cytological material is obtained using both conventional and liquid-based methods; the second occurs in the anatomical pathology laboratory, where the sample is prepared on a slide and examined under a microscope (Fig. 1). The simulation was supervised by the same female professor and medical pathologist who had taught the theoretical class.

### Method of data collection

Emotional responses were collected in April 2025 using a structured questionnaire based on the 13 categories of the Pleasure Framework proposed by Costello and Edmonds [6]: Creation, Exploration, Discovery, Difficulty, Competition, Danger, Captivation, Sensation, Empathy, Simulation, Fantasy, Camaraderie, and Subversion. The Sensation category referred to physical stimuli (e.g., movements, vocalizations, tactile or auditory experiences). Each category was assessed using a modified Self-Assessment Manikin (SAM) instrument [7], with responses recorded on a six-point (0–5) ordinal response scale. Students were also encouraged to provide written justifications for their scores, enabling qualitative insights [8].

#### Step 1. Theoretical preparation

Prior to the simulation, students attended a 60-minute theoretical class on gynecological cytology. The class, taught by a female professor and medical pathologist, aimed to introduce key concepts related to the collection, preparation, and analysis of cytological slides [9].

#### Step 2. Digital simulation

Students were guided through the PapSim digital simulator in the university’s computer lab. The professor remained present to offer technical and scientific support. Each group underwent the simulation for approximately 25 to 60 min.

#### Step 3. Emotional evaluation

Immediately after completing the simulation, students filled out the emotional evaluation questionnaire [8]. The questionnaire included six visual response options represented by facial expressions ranging from “I felt very little” (0) to “I felt very much” (5), together with open-ended questions for written feedback. This step captured both quantitative ratings and qualitative perceptions of the experience.

### Statistical and qualitative analysis

Quantitative data obtained from the six-point ordinal response scales were analyzed using R software. For each of the 13 categories of the Pleasure Framework, descriptive statistics were calculated to obtain the mean values. Subsequently, Spearman’s rank correlation coefficient (ρ) was used to examine the relationships between emotional dimensions, with statistical significance set at *p* < 0.05. This non-parametric test was chosen based on the ordinal nature of the data and the sample size. The correlation matrix helped identify emotional coherence and possible interconnections among cognitive, affective, and social elements in the students’ learning experience.

Qualitative analysis was performed by examining the open-ended responses provided by participants. A content analysis was conducted for each of the 13 categories of the Pleasure Framework. Responses were grouped thematically, and both absolute and relative frequencies were calculated. The three categories that received the highest number of justifications, namely Exploration, Discovery, and Simulation, were selected for detailed analysis because they generated the largest number of qualitative responses and represented the emotional dimensions most frequently discussed by participants. The findings are presented in Tables 2, 3, and 4, respectively. This approach enabled a comprehensive understanding of students’ subjective experiences and emotional engagement during the simulation.

To promote transparency and support reproducibility, the programming documentation and implementation files of the PapSim prototype have been deposited in the Zenodo repository (DOI: 10.5281/zenodo.19367490), allowing other researchers to access the material.

## Results

The study generated both quantitative and qualitative data regarding students’ emotional responses. While the following section presents the quantitative results, the written justifications also provided valuable insights into students’ perceptions of the learning experience and their evaluation of the simulator.

### Quantitative results

Based on sociodemographic data voluntarily provided by second-year nursing students from Class 85 at UNIFESP, 37 out of 40 participants reported their sex and age, and 36 disclosed their academic performance (GPA). The majority of respondents were female (*n* = 34), with only three male students. Participants ranged in age from 18 to over 35 years, with the largest proportion belonging to the 21–23-year age group (*n* = 13). Regarding academic performance, the GPA values reported indicated generally strong academic achievement, ranging from 7.300 to 9.490 (Table 1). The voluntary nature of data collection may account for the incomplete dataset. Nonetheless, the results reflect a student profile predominantly composed of young women with high academic performance, which may have contributed to their engagement with innovative teaching methods such as digital simulation.

**Fig. 2:**
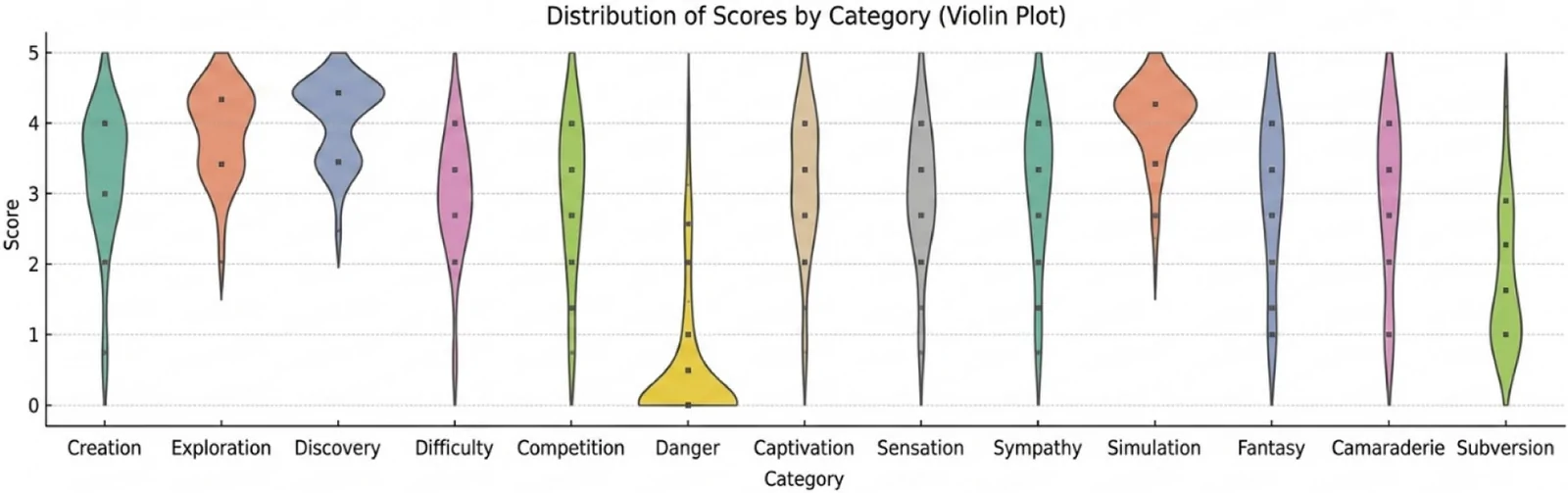
Distribution of students' emotional response scores across the 13 categories of the Pleasure Framework [6]. The plot illustrates the distribution and density of responses on the six-point (0–5) ordinal response scale. Higher concentrations of responses were observed in the Simulation, Discovery, and Exploration categories, whereas the Danger category showed predominantly low scores, indicating a low perceived sense of risk or discomfort during the simulation

The violin plot further emphasizes the distribution shape of student responses, revealing density peaks in the upper range for Creation, Exploration, and Simulation, reflecting frequent reports of positive emotional states. Conversely, Danger exhibited a pronounced skew toward the lowest scale values, corroborating the limited perception of risk or discomfort during the activity. These visualizations indicate high levels of learner engagement and positive emotional responses, particularly in categories aligned with creativity, discovery, and immersive simulation, while also identifying areas where emotional activation was less evident (Fig. 2).

The highest mean scores were observed in the categories of Simulation (4.625), Discovery (4.600), and Exploration (4.475), suggesting that students experienced these dimensions most intensely and positively during the simulation. These results indicate high levels of engagement and perceived realism, supporting the acceptability of the simulator as an immersive educational tool. Conversely, the lowest scores were recorded in Danger (0.775) and Subversion (1.225), suggesting that students did not perceive the experience as risky or unconventional - a finding consistent with the characteristics of a safe and structured educational environment. Intermediate mean values were found in categories such as Competition (3.350), Camaraderie (3.300), and Fantasy (3.300), reflecting more variable experiences among participants, possibly influenced by individual interpretations or prior expectations within the simulation context. The results related to competition and camaraderie may suggest that the simulator was primarily experienced as an individual learning activity. The highest standard deviations were observed in Camaraderie (1.800) and Fantasy (1.713), indicating a broader range of emotional responses, which may reflect the subjective nature of these categories or differing levels of social and imaginative engagement among students. The findings suggest that the PapSim digital simulator was positively received, particularly in terms of Simulation, Discovery, and Exploration. The low levels of perceived danger and subversion indicate that participants experienced the activity as safe and appropriate within the educational context, supporting its use as an interactive pedagogical tool in health education (Table 1).

**Table 1:** Demographic characteristics and academic performance of study participants (*n* = 37)

Sex	Age range (years)	GPA
F	24–26	8.903
F	24–26	8.558
F	21–23	8.423
F	21–23	8.536
F	21–23	8.391
F	18–20	8.383
F	18–20	8.835
F	21–23	8.241
F	18–20	8.498
F	24–26	8.261
F	24–26	8.795
F	21–23	8.565
F	> 35	Missing
F	21–23	9.030
F	27–35	8.480
F	21–23	8.598
F	21–23	7.894
F	21–23	8.999
F	18–20	8.407
F	18–20	9.000
F	21–23	8.300
F	18–20	8.216
M	18–20	8.056
F	24–26	7.642
F	21–23	8.680
F	18–20	7.422
F	24–26	9.167
F	18–20	8.953
F	18–20	8.434
F	18–20	8.866
F	24–26	8.400
F	27–35	8.390
M	24–26	8.800
M	21–23	8.079
F	21–23	8.234
F	27–35	9.490
F	27–35	7.300

*Legend: F *Female, *M *Male, *GPA *Grade Point Average

**Fig. 3:**
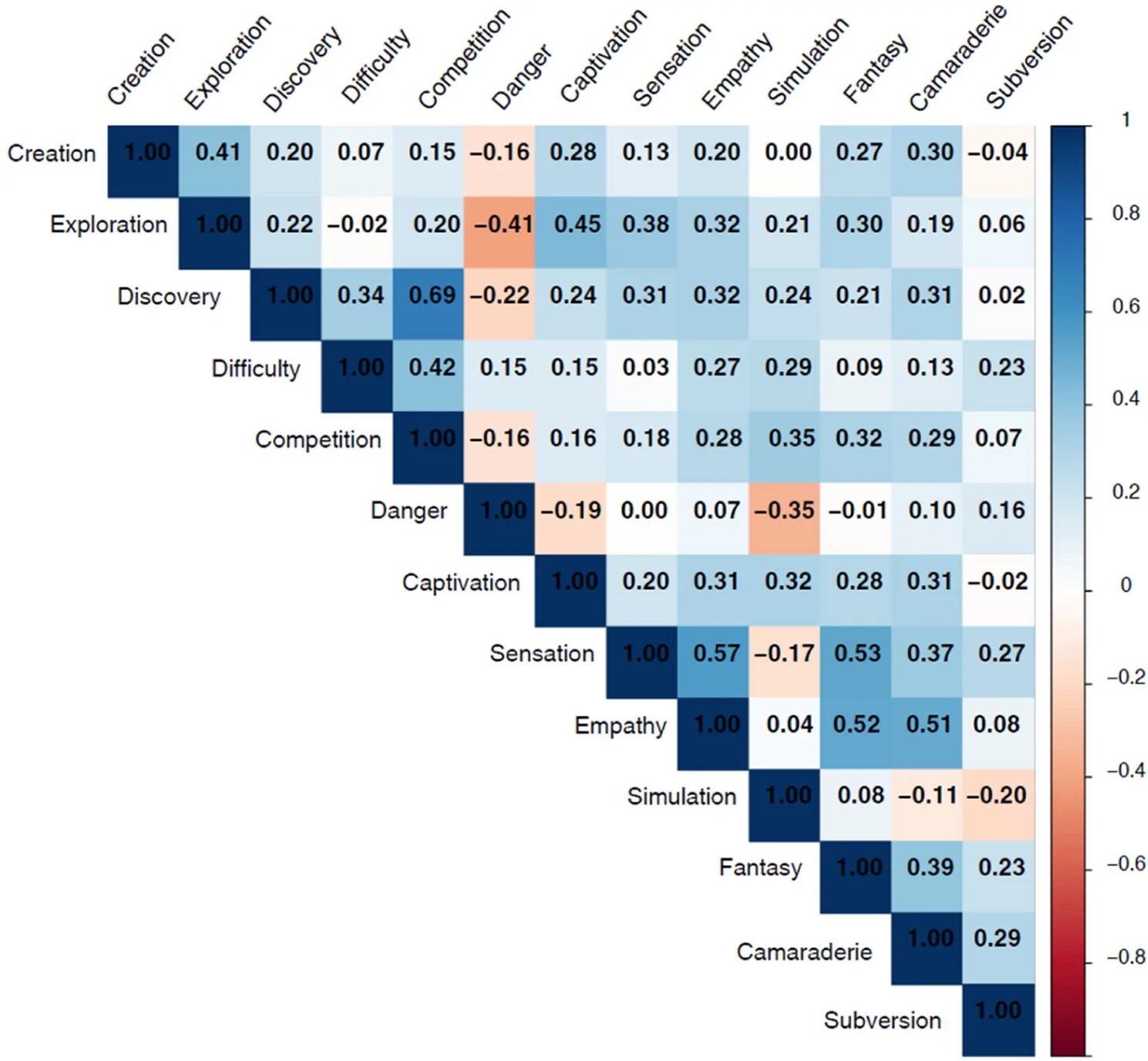
Spearman correlation matrix of emotional response categories. The matrix displays Spearman’s correlation coefficients among the 13 emotional categories of the Pleasure Framework [6]. Strong positive correlations were observed between Discovery and Competition, as well as between Sensation, Empathy, and Fantasy, indicating emotional coherence in students’ experiences. In contrast, Danger showed weak or negative correlations, reinforcing the perception of a safe and non-threatening environment

The Spearman correlation analysis across the 13 Pleasure Framework categories, as outlined by Costello and Edmonds [6], revealed significant associations among several emotional dimensions experienced during the use of the PapSim simulator. The strongest and statistically significant correlation was found between Discovery and Competition (ρ = 0.69, *p* < 0.001), suggesting that the sense of discovery was strongly associated with task resolution and challenge-oriented engagement. Additionally, strong positive correlations were observed between Sensation and Empathy (ρ = 0.57, *p* < 0.001), Sensation and Fantasy (ρ = 0.53, *p* < 0.001), Empathy and Fantasy (ρ = 0.52, *p* < 0.001), and Empathy and Camaraderie (ρ = 0.51, *p* < 0.001). These findings indicate that positive sensory experiences were closely associated with empathy, interpersonal involvement, and immersion in the simulated environment (Fig. 3).

### Qualitative results

The analysis of the justifications provided by the 40 students who participated in the PapSim simulation revealed a rich diversity of emotional, cognitive, and social perceptions, categorized according to the 13 domains of the Pleasure Framework [6]. Not all students provided written justifications, which explains the variation in the number of responses across categories. After coding and categorizing the responses, the findings were summarized in Tables 2, 3 and 4.

**Table 2:** Exploration: thematic content analysis of participants’ responses

Interpretative Category	No. Responses	Frequency (%)	Example Quotes
Exploration of instruments and technical environments	10	37.0%	“The simulator allows exploring all the examination steps.”
Discovery-based learning and curiosity	6	22.2%	“I loved discovering what each instrument did.”
Playful, enjoyable, or engaging experience	4	14.8%	“It was fun exploring the topic through the simulator.”
General positive comment on learning	3	11.1%	“It was very good to learn new things with this simulator.”
Criticism of repetition or game rigidity	2	7.4%	“I wish I could interact without following a strict order.”
Neutral or ambiguous comment	2	7.4%	“The game seems to be halfway through its development.”
Total Responses	27	100%	

**Table 3:** Discovery: thematic content analysis of participants’ responses

Interpretative Category	No. Responses	Frequency (%)	Example Quotes
Clinical diagnosis and problem-solving	11	40.7%	“I liked getting the diagnosis right”; “Making a diagnosis”
Technical knowledge (process, slides, analysis)	7	25.9%	“I liked understanding the histological exam process”
Curiosity and discovery-based learning	5	18.5%	“It was interesting to discover how the process happens”
Didactics and clarity of explanation	2	7.4%	“Very well-explained content”
General or emotional positive comment	2	7.4%	“Satisfying, to say the least”; “Surprise”
Total Responses	27	100%	

**Table 4:** Simulation: thematic content analysis of participants’ responses

Interpretative category	No. responses	Frequency (%)	Example quotes
Realism and connection with professional practice	11	40.7%	“It simulates real life”; “Very realistic”; “I felt what the routine is like for those who work in this area”
Interaction with laboratory and instruments	6	22.2%	“I felt like I was in the lab”; “I enjoyed how I interacted with the lab”
Understanding of technical processes (step-by-step)	5	18.5%	“The simulator helps us understand each part of the process”; “The step-by-step is helpful”
Immersion and sense of belonging	3	11.1%	“Immersive”; “Nice to feel part of it”
General comments or remarks on development potential	2	7.4%	“Still under development”; “Even as a prototype, it already shows potential”
Total responses	27	100%	

Spontaneous social interaction emerged as a notable finding, despite the simulator being initially designed for individual use. Students from both groups (27 and 13 participants) reported engaging in discussions about procedures, diagnostic reasoning, and instrument handling during the simulated scenarios. This emergent behavior suggests that PapSim, although digital and guided, fostered socio-enactive and collaborative learning experiences.

The most frequently cited and positively evaluated categories were Simulation, Discovery, Exploration, and Creation. Students highlighted the technical realism, the opportunity to participate in all stages of the examination, the sense of discovery when identifying diagnoses, and the autonomy in navigating the simulator’s environments. These perceptions indicate strong emotional engagement and support the acceptability of PapSim as an educational resource for teaching gynecological cytology.

The Difficulty category was perceived as moderate and appropriate to students’ knowledge level, with some suggesting the inclusion of more advanced challenges. Captivation showed a high degree of engagement, although some comments mentioned informational overload, which could be addressed in future versions.

In the Competition category, most students did not perceive direct competitive elements but valued the achievement of individual goals as a motivational factor. Conversely, the Danger and Subversion categories received minimal responses, indicating that the digital environment was perceived as safe and appropriate for educational use. The limited perception of transgression was consistent with the simulator’s educational purpose.

The Sensation and Empathy categories revealed that visual stimuli were the most valued, with suggestions for the inclusion of sounds and greater physical interactivity. Empathy primarily emerged in relation to the patient and instructor characters, although some students reported difficulty in establishing emotional connections, suggesting room for narrative improvement.

The Fantasy category was interpreted mainly as an extension of realism, with emphasis on the playful effect and immersive feeling. In Camaraderie, opinions were divided: while some students reported a connection with the characters and instructors, others did not perceive emotional bonds, possibly due to the short duration of the experience.

Lastly, the additional comments revealed constructive suggestions for improving visual design, usability, and technical content. Students demonstrated critical thinking by proposing greater navigational freedom, the possibility of content review, and adjustments to graphic elements.

These qualitative findings reinforce students’ positive perceptions of PapSim, particularly regarding emotional engagement, realism, and reflection on the educational experience. Even in its prototype stage, the simulator was well received and demonstrated potential to evolve as an innovative and complementary educational tool for teaching gynecological cytology.

## Discussion

The methodological choice to apply the evaluation only after the simulation aligns with the approach used by Abdulmohdi and McVicar [10], who adopted a descriptive qualitative design focused exclusively on student perceptions following the simulated experience. In that study, semi-structured interviews were conducted to explore how students perceived the contribution of simulation to clinical decision-making. Likewise, the present study employed the Pleasure Framework instrument following the PapSim experience, with a focus on analyzing the emotional and cognitive dimensions elicited during the activity. This strategy proved effective in capturing affective impressions, critical reflection, and experiential engagement, particularly in the context of prototype validation.

The results of the PapSim application suggest the potential of digital simulation as a complementary educational tool for teaching gynecological cytology [5]. High mean scores in categories such as Simulation, Discovery, and Exploration indicate that the prototype offered a meaningful and realistic learning experience, fostering emotional engagement and positive perceptions among nursing students. These findings are consistent with Hipólito et al., [4], who reported positive student responses to serious games designed for learning oncotic cytology collection.

The qualitative findings further support the quantitative data by highlighting students’ engagement with the PapSim prototype across emotional, cognitive, and social dimensions. As summarized in Tables 2, 3 and 4, the categories Simulation, Discovery and Exploration stood out, emphasizing students’ perceptions of the simulator as a realistic and interactive educational experience. While the number of responses varied across the 13 Pleasure Framework categories, the data revealed a wide range of emotional experiences, providing evidence of active engagement and reflective learning during the simulation. Categories like “Danger” and “Subversion” received minimal mentions, confirming the simulator’s perception as safe and appropriate for the educational context.

The Spearman correlation analysis revealed patterns of emotional coherence among the most significant dimensions. The correlation between Discovery and Competition indicates that students who felt challenged to solve tasks also reported a strong sense of discovery. This association suggests that problem-solving and cognitive engagement were closely tied to perceptions of individual progress and achievement, which are central elements in active learning processes.

The relationship between Sensation and Empathy suggests that sensory stimuli, including visual, tactile, and simulated were strongly connected to feelings of empathy or emotional closeness with characters or the simulation setting. Pleasant sensory experiences may have contributed to affective identification. Similarly, the correlation between Sensation and Fantasy indicates that sensory elements also supported creative immersion. Even though the simulator was grounded in clinical reality, students used imagination to project themselves into the scenes and experience the simulated context more deeply.

The association between Empathy and Fantasy shows that empathy generated by the characters or storyline encouraged engagement with the more playful narrative elements. Emotional connection with the simulated cases may have encouraged students’ creative involvement. Furthermore, the correlation between Empathy and Camaraderie points to the influence of empathy on spontaneous collaboration. Students exchanged information about clinical cases and procedures, which may have been encouraged by their emotional connection to the characters and the setting [11]. This behavior reflects socio-enactive learning, as discussed by Imamura and Baranauskas [12], and suggests opportunities for collaborative knowledge construction. Such engagement is also influenced by the broader learning environment, as Detyna et al. [13] argue that student interaction and participation are shaped not only by content but also by the socio-material configuration of the classroom space.

The study by Koivisto et al. [11], conducted with 52 nursing students from three Finnish universities, aimed to explore both user experience and empathy in an immersive virtual reality simulation. The game was developed using the Unity platform, as was PapSim. However, Koivisto’s study used a pre- and post-simulation assessment model, allowing the authors to observe increased empathy levels during training. The authors reported increased empathy scores following the simulation, which were associated with more empathetic professional behaviors in student-patient relationships. The authors also found correlations between user experience and aspects of empathy, such as affective sharing and cognitive empathy [11]. A key element was the simulation’s emphasis on the student playing the role of the nurse, not the patient. This role immersion stimulated helping behavior, empathetic concern, and motivation. Similarly, PapSim placed students in an active caregiver role, covering sample collection and laboratory analysis. Although empathy was not measured before and after the simulation in the present study, the observed correlations in the Pleasure Framework, specifically between Sensation and Empathy, Fantasy and Empathy, and Camaraderie and Empathy, suggest emotional closeness to the simulation setting and characters. Even though the simulator was designed for individual use, students engaged in spontaneous exchanges about clinical scenarios, suggesting that it may encourage collaborative interactions during the learning experience.

The findings of this study align with those of Mäkinen et al. [14], who explored user experience among 41 nursing students in a virtual reality simulation. Participants noted that pre-set choices limited realism and that allowing more autonomy in clinical decisions would lead to increased satisfaction. This observation was echoed by PapSim users, who suggested that greater realism enhances engagement. Additionally, Mäkinen’s study reported student concerns about the overuse of computer-based education, suggesting that simulations should supplement rather than replace traditional teaching methods. In this sense, PapSim already operates as a complementary tool, reinforcing theoretical content prior to clinical training. Moreover, participants in Mäkinen’s study highlighted the value of rehearsing stressful situations in a safe environment. Similarly, participants in the present study perceived PapSim as a safe educational environment, suggesting that it may serve as a useful preparatory resource before clinical activities in the Women’s Health course [10].

When compared to other simulation-based educational strategies, such as the Histopoly game described by Marcos et al. [15], it becomes clear that gamification and realism play a central role in motivating students and promoting content retention. Similarly, studies by Owens et al. [16] and Durgun et al. [17] emphasize that low-cost simulators are effective training tools, even in low-resource educational contexts.

Students’ suggestions to implement the simulator prior to the Women’s Health course emphasize its potential as a preparatory tool, facilitating early familiarization with technical procedures and promoting student autonomy.

Taken together, these findings suggest that PapSim, even as a prototype, was positively perceived as supporting emotional engagement and collaborative interaction during the learning experience. These findings support its potential as a complementary educational tool in nursing education and are reinforced by evidence from similar simulations, as well as by students’ constructive feedback regarding usability, design, and immersive qualities.

### Limitations of the study

Although the findings of this study indicate positive student acceptance and pedagogical potential for the PapSim simulator, some limitations should be acknowledged. The sample consisted of only 40 nursing students, which restricts the generalizability of the findings to other educational contexts. Additionally, the simulator was applied to a single cohort (Class 85 from UNIFESP), limiting variability in academic backgrounds and institutional settings that could influence the outcomes. Another relevant aspect is the absence of a control group using traditional methods or alternative tools, which prevents a direct attribution of the observed effects solely to the use of PapSim.

This study assessed students’ perceptions and experiences with the PapSim prototype. Future studies incorporating objective educational outcome measures may provide additional evidence regarding its educational value in nursing education.

## Conclusions

In summary, the PapSim prototype was positively perceived and well accepted by nursing students as an engaging, realistic, and interactive complementary educational resource for teaching gynecological cytology. These findings highlight the potential of digital simulation to complement the teaching of gynecological cytology and provide a foundation for the continued refinement and evaluation of the PapSim prototype. Future studies involving larger and more diverse samples, together with objective educational outcome measures, are warranted to further evaluate its educational value and broader applicability in nursing education.

## Data Availability

The datasets generated and analyzed during the current study are available from the corresponding author upon reasonable request.

## Abbreviations

HPV: Human Papillomavirus
ICF: Informed Consent Form
SAM: Self-Assessment Manikin
GPA: Grade Point Average
CAPES: Coordination for the Improvement of Higher Education Personnel
CAAE: Brazilian research ethics approval registration number
UNIFESP: Universidade Federal de São Paulo
